# Effects of Single Amino Acid Substitution on the Biophysical Properties and Biological Activities of an Amphipathic α-Helical Antibacterial Peptide Against Gram-Negative Bacteria

**DOI:** 10.3390/molecules190810803

**Published:** 2014-07-24

**Authors:** Juanjuan Tan, Jinfeng Huang, Yibing Huang, Yuxin Chen

**Affiliations:** 1 Key Laboratory for Molecular Enzymology and Engineering of the Ministry of Education, Jilin University, Changchun 130012, China; 2 National Engineering Laboratory for AIDS Vaccine, Jilin University, Changchun 130012, China; 3 School of Life Sciences, Jilin University, Changchun 130012, China; 4 School of Life Sciences, Northeast Normal University, Changchun 130012, China

**Keywords:** antimicrobial peptide, hydrophobicity, biological activity, Gram-negative bacteria, mechanism of action

## Abstract

An antimicrobial peptide, known as V13K, was utilized as the framework to study the effects of charge, hydrophobicity and helicity on the biophysical properties and biological activities of α-helical peptides. Six amino acids (Lys, Glu, Gly, Ser, Ala, and Leu) were individually used to substitute the original hydrophobic valine at the selected sixteenth location on the non-polar face of V13K. The results showed that the single amino acid substitutions changed the hydrophobicity of peptide analogs as monitored by RP-HPLC, but did not cause significant changes on peptide secondary structures both in a benign buffer and in a hydrophobic environment. The biological activities of the analogs exhibited a hydrophobicity-dependent behavior. The mechanism of peptide interaction with the outer membrane and cytoplasmic membrane of Gram-negative bacteria was investigated. We demonstrated that this single amino acid substitution method has valuable potential for the rational design of antimicrobial peptides with enhanced activities.

## 1. Introduction

The growing problems of antibiotic resistance threaten human health, due to the fact that there are few antibiotics against resistant bacteria in the pipeline [1,2,3]. Among bacteria, Gram-negative bacteria are considered as more difficult to deal with in clinical practice [1,2]. As a category of promising molecules, antimicrobial peptides (AMPs) have been widely investigated for their outstanding properties, such as rapid action, broad killing spectrum and difficulty in evoking resistance. Naturally existing AMPs are highly variable and most AMPs are short cationic peptides with 12–100 amino acids [4,5]. The mechanism of action of AMPs has been widely investigated, and two main modes have been accepted: membrane disruption and non-membrane disruption. The first one involves the electrostatic and hydrophobic interactions of peptides and phospholipid molecules of membrane and the latter manner involves the interaction of peptides with cytoplasmic anionic molecules [4,6].

In the proposed mechanism of action, the bactericidal processing of cationic AMPs occurs in a stepwise manner and is generally rapid against both Gram-positive and Gram-negative bacteria [4,7]. The first step of the mechanism involves the migration of the AMPs toward the cell membrane due to electrostatic forces [8]. Both the cell wall of Gram-negative or Gram-positive bacteria and the inner cytoplasmic membrane of Gram-negative bacteria contain negatively charges and contribute to the electrostatic attraction of the cationic peptide [9]. The outer surface of the cell wall of Gram-negative bacteria is known to be composed of negatively charged lipopolysaccharides (LPSs), which make this type of bacteria the toughest to deal with in clinical practices. To reach the cytoplasmic membrane target, peptides first pass through LPS. AMPs destroy the outer membrane of Gram-negative bacteria by displacement of the divalent cations, such as Mg^2+^ and Ca^2+^, which stabilize the LPS by binding to the anionic phosphate groups, then AMPs target the inner membrane and destroy the inner membrane by making holes or channels [5]. Initially, cationic peptides contact with the negatively charged lipid head groups on the surface of the membrane, and an amphipathic conformation will be induced for most α-helical AMPs. During this folding process, it is proposed that AMPs adopt a parallel orientation to the membrane [4]. Among the models to explain the permeabilizing process, the carpet model, barrel-stave model and toroidal-pore model are widely accepted. Recently, based on the “barrel-stave” model [10] and the “carpet” model [11], Chen *et al.* proposed a “membrane discrimination” model for AMPs whose sole target is the biomembrane [12,13]. Prokaryotic and eukaryotic cell membranes have different structures and functions. Prokaryotic membranes have a high negative net charge and are predominantly composed of phosphatidylglycerol (PG), cardiolipin (CL), or phosphatidylserine (PS). In contrast, mammalian membranes are enriched in the zwitterionic phospholipids (neutral net charge) phosphatidylethanolamine (PE), phosphatidylcholine (PC) or sphingomyelin (SM) [9,14]. Moreover, the mammalian cell membrane contains cholesterol [15], it has been reported that cholesterol can dramatically reduce the activity of AMPs by stabilizing the lipid bilayer or by directly interacting and neutralizing AMPs [16].

The major barrier to the application of antimicrobial peptides as antibiotics is their toxicity or ability to lyse eukaryotic cells. This is perhaps not a surprising result if the target is indeed the cell membrane [17,18]. Hence, enhancing selectivity is crucial for the clinical application of AMPs. As the most widely distributed AMPs in nature, α-helical antimicrobial peptides have been thoroughly investigated. In general, the antimicrobial activity and hemolytic activity in amphipathic α-helical antimicrobial peptides are related to multiple physicochemical parameters, including peptide length, sequence, charge, helicity, hydrophobicity, amphipathicity, hydrophobic/hydrophilic angle and self-association [8]. We believe that a synthetic peptide approach to examining the effect of small incremental changes in the physicochemical parameters of cationic antimicrobial peptides will enable rapid progress in the rational design of peptide antibiotics. In this study, we systematically modulated the peptide biophysical properties of the parent peptide by single amino acid substitutions to explore the *de novo* approach of peptide design with better selectivity. The antibacterial mechanism of action against Gram-negative bacteria was also investigated with the change of peptide biophysical properties.

## 2. Results and Discussion

### 2.1. Peptide Design and the Biophysical Properties

**Figure 1:**
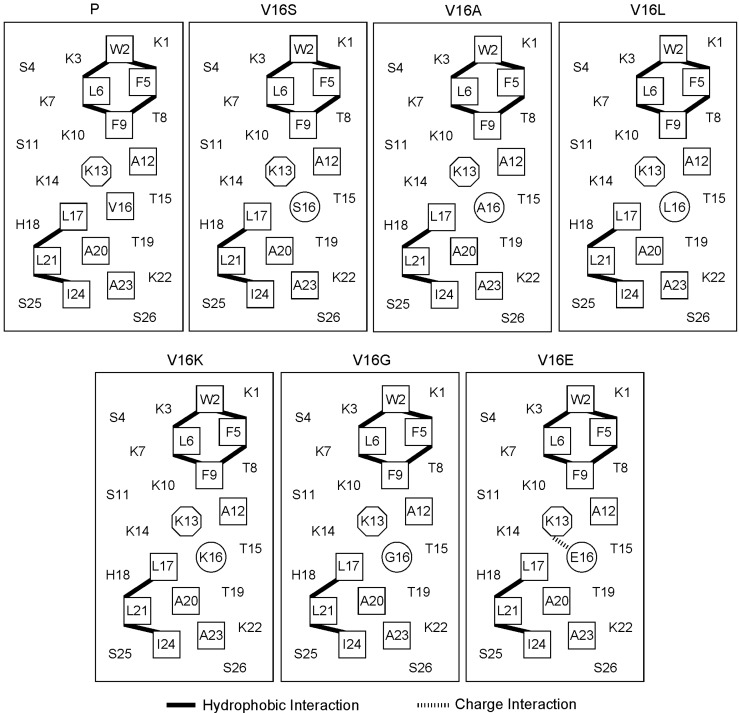
Helical net representation of peptides analogs. The hydrophobic amino acid residues on the non-polar faces are boxed. The substituting amino acids on the non-polar face are circled. The lysine at the position 13 on the non-polar face is in octagon. The *i* → *i* + 3 and *i* → *i* + 4 hydrophobic interactions and charge interactions are shown as black bars and hashed bars, respectively. The one-letter code is used for the amino acid residues.

The parent peptide (peptide P) (Figure 1), also known as peptide V13K, is an amphipathic AMP, which will be induced into an α-helical secondary structure with a defined polar face and a non-polar face upon a hydrophobic environment as reported previously [12]. In the present study, we selected the sixteenth valine residue on the non-polar face as the substitution site and systematically replaced valine with six other amino acids (lysine, glutamic acid, serine, glycine, alanine, leucine) with different hydrophobicity. The amino acid sequences of the analogs are shown in Table 1. 

As the central location substitutions had great effects on the properties of peptides [12,19], the amino acid substitutions at the sixteenth position should maximize the effect of single amino acid substitutions. It is noteworthy that, the *i* → *i* + 3 and *i* → *i* + 4 hydrophobic interactions were not changed upon the amino acid substitutions at this location; in contrast, the *i* → *i* + 3 and *i* → *i* + 4 charge interactions were altered by introducing Glu and Lys (Figure 1).

Single amino acid substitutions showed dramatic effects on the peptide hydrophobicity, as determined by the RP-HPLC retention times (Table 2). Among substitutions, the retention times ranged from 32.2 min (peptide V16K) to 40.4 min (peptide V16L), which reflect the pure effects of single amino acid substitutions. Thus, the retention times of peptides reflect the relative hydrophobicity of side chains. Consistent with previous studies [20], the hydrophobicity of peptide analogs was generally in the following order: Lys < Gly < Ser < Ala < Val < Leu. Glu-substituted peptide was an exception, as it contains a negatively-charged side chain and this should decrease the hydrophobicity of the peptide dramatically. In fact, the hydrophobicity of peptide V16E was comparable with peptide V16S, which may be attributed to the fact that the electrostatic interactions between K13 and E16 may reinforce the peptide secondary structure and result in a higher relative hydrophobicity (Figure 1).

**Table 1:** Sequence of peptides used in this study.

Peptide	Amino Acid Sequence *^a^*
P	Ac-K-W-K-S-F-L-K-T-F-K-S-A-K-K-T-V-L-H-T-A-L-K-A-I-S-S-amide
V16K	Ac-K-W-K-S-F-L-K-T-F-K-S-A-K-K-T-*K*-L-H-T-A-L-K-A-I-S-S-amide
V16G	Ac-K-W-K-S-F-L-K-T-F-K-S-A-K-K-T-*G*-L-H-T-A-L-K-A-I-S-S-amide
V16S	Ac-K-W-K-S-F-L-K-T-F-K-S-A-K-K-T-*S*-L-H-T-A-L-K-A-I-S-S-amide
V16E	Ac-K-W-K-S-F-L-K-T-F-K-S-A-K-K-T-*E*-L-H-T-A-L-K-A-I-S-S-amide
V16A	Ac-K-W-K-S-F-L-K-T-F-K-S-A-K-K-T-*A*-L-H-T-A-L-K-A-I-S-S-amide
V16L	Ac-K-W-K-S-F-L-K-T-F-K-S-A-K-K-T-*L*-L-H-T-A-L-K-A-I-S-S-amide

*^a^* One-letter codes are used for the amino acid residues; Ac, N^α^-acetyl; amide, C-terminal amide; the bold and italic letters denote the substituting amino acids of the peptide P. All amino acids are L-amino acids.

**Table 2:** Biophysical data of the peptide analogs.

Peptide *^a^*	*t*_R_(min) *^b^*	Benign *^c^*		50%TFE *^d^*	
		[*θ*]_222_	% helix *^e^*	[*θ*]_222_	% helix *^e^*
V16K	32.2	−1,990	6.6	−29,313	97.5
V16G	33.6	−2,839	9.4	−27,857	92.7
V16S	34.3	−1,760	5.9	−28,949	96.3
V16E	34.6	−3,465	11.5	−30,063	100.0
V16A	36.6	−3,057	10.2	−28,769	95.7
P	38.2	−2,592	8.6	−29,726	98.9
V16L	40.4	−3,595	12.0	−29,803	99.1

*^a^* Peptides are ordered by relative hydrophobicity during RP-HPLC at 25 °C. *^b^* Retention times of peptides during RP-HPLC at 25 °C. *^c^* The mean residue molar ellipticities, [*θ*]_222_(degree·cm^2^· dmol^−^^1^) at wavelength 222 nm were measured at 25 °C in KP bsuffer (100 mM KCl, 50 mM K_2_HPO_4_/KH_2_PO_4_, pH 7.0). *^d^* The mean residue molar ellipticities, [*θ*]_222_(degree·cm^2^·dmol^−^^1^) at wavelength 222 nm were measured at 25 °C in KP buffer diluted 1:1 (v/v) with TFE. *^e^* The helical content (in percentage) of a peptide relative to the molar ellipticity value of peptide V16E in 50% TFE.

**Figure 2:**
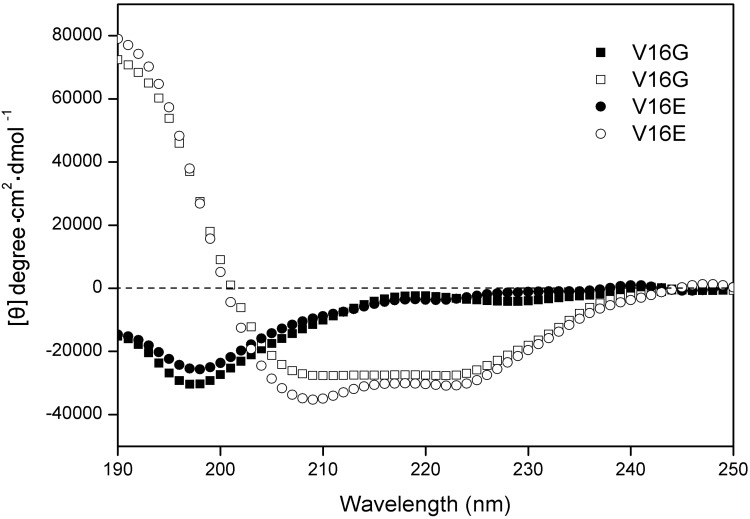
CD spectra of peptide V16G and V16E at pH 7 and 25 °C in 50 mM KH_2_PO_4_/K_2_HPO_4_ containing 100 mM KCl. Solid symbols denote the CD spectra of peptide analogs in KP buffer without TFE, and open symbols denote CD spectra obtained in the presenceof 50% TFE. Peptide V16G data are presented by squares and V16E by circles.

The secondary structures of peptides were determined in benign buffer and in a hydrophobic environment (50% trifluoroethanol, TFE) to mimic the hydrophobic environment of the membrane. As shown in Table 2, all peptides exhibited random coil structures in the aqueous environment and were induced into a fully helical structure in the presence of 50% TFE. According to a previous study [21], the α-helical propensity of the seven amino acids used in this study follows the order: A > L > K > V > S > E > G, which indicated that peptides V16E and V16G might exhibit smaller α-helical content compared to other peptides. The principle of side chain interaction at the *i* to *i* + 3 and *i* to *i* + 4 positions has been used to promote α-helix stability [22], for example, the incorporation of Lys and Glu residues to stabilize electrostatic attractions [23]. Thus, the helicity of peptide V16E in the presence of 50% TFE that presented the greatest value among all analogs should rely on the contribution of the electrostatic attraction of the side chain of K13 and E16, which stabilizes the helix. The relative helicity is 100% for peptide V16E in 50% TFE, showing the importance of the salt bridges of K13 and E16 to stabilize the helical structure. In general, subtle side-chain alterations by single amino acid substitutions made no significant changes on secondary structures both in aqueous and in hydrophobic environments (Table 2). The CD spectra of peptide V16G and V16E are shown in Figure 2.

### 2.2. Biological Activities of Peptides Analogs

The antimicrobial activity of the peptide analogs was determined against five Gram-negative bacterial standards and clinic strains, as shown in Table 3. The geometric mean values were used to evaluate the overall antimicrobial activity of peptides. It is clear that the antimicrobial activities of the peptides had a correlation with the peptide hydrophobicity, *i.e.*, the more hydrophobic the peptide, the more active it is against bacteria, which is consistent with a previous study [13]. However, the introduction of the negatively-charged amino acid glutamic acid decreased the antimicrobial activity dramatically, *i.e.*, the MIC value of V16L against *P. aeruginosa* decreased from 2 μg/mL to 64 μg/mL when a Glu-substitution was introduced (peptide V16E, Table 3). Compared with the negative-charged residue substituted analog, V16K showed much better antimicrobial activity in general against Gram-negative bacteria, which reinforced the importance of a net positive charge for the peptide antimicrobial activity.

**Table 3:** Minimal inhibitory concentration (MIC) of peptides against Gram-negative bacteria and hemolysis percentage against human red blood cells.

Peptide *^a^*	MIC (μg/mL)					Hemolysis Percentage *^b^*	GM *^c^*
	*E.* *coli* ATCC25922	*E.* *coli* DH5*α*	*E.* *coli* Clinical Isolate	*P.* *aeruginosa* ATCC27853	*P.* *aeruginosa* H188		
V16K	32	16	8	32	16	6.2	18.4
V16G	8	8	16	32	8	8.1	12.1
V16S	8	8	16	32	4	11.3	10.6
V16E	16	64	125	125	64	3.9	63.4
V16A	2	4	1	4	4	14.3	2.6
P	1	2	1	4	4	28.3	2.0
V16L	1	4	1	2	2	53.9	1.7

*^a^* Peptides are ordered by relative hydrophobicity during RP-HPLC at 25 °C. *^b^* Hemolysis percentage was determined at the peptide concentration of 1,000 μg/mL against human red blood cells. *^c^* GM denotes the geometric mean of MIC values from all five Gram-negative bacterial strains in this table.

**Figure 3:**
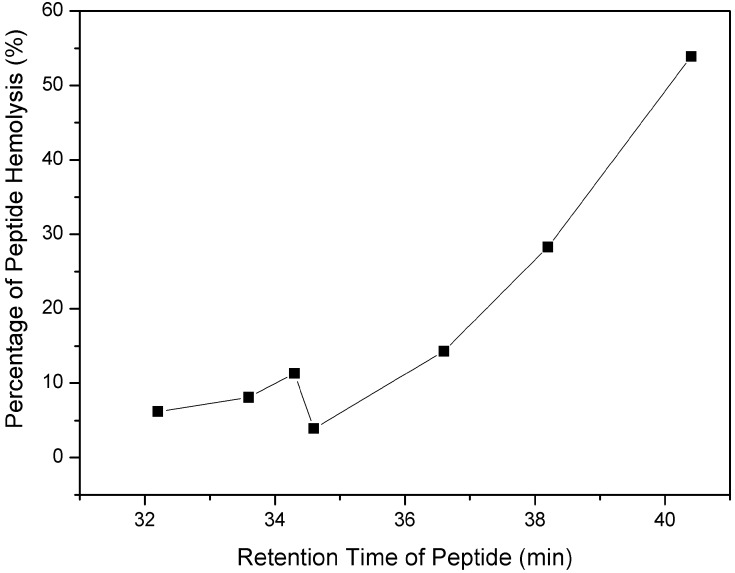
Relationships of peptide hydrophobicity and hemolytic activity. Hydrophobicity was expressed as the retention times of peptides in RP-HPLC at room temperature. Hemolytic activity was assessed by the percentage of peptide hemolysis at 1,000 μg/mL.

The toxicity of the peptide analogs to the eukaryocyte was assessed against human red blood cells. In general, all peptide analogs exhibited little or negligible hemolytic activity. The hemolysis percentage of peptides at 1,000 μg/mL was plotted against the RP-HPLC retention time of the peptides (Figure 2). It is clear that the hemolytic activity of the peptides was correlated with peptide hydrophobicity as shown in Figure 3, that is, peptides with higher hydrophobicity generally exhibited stronger hemolytic activity against red blood cells. 

### 2.3. Outer Membrane Permeabilization and LPS Binding Affinity

For the membrane system of Gram-negative bacteria, there is an outer membrane within the cell wall, thus, AMPs first act on the outer membrane. In this study, 1-*N*-phenylnaphthylamine (NPN) uptake assay was used to assess the effects of substitutions on the outer membrane permeabilization. The fluorescence character of NPN, a small hydrophobic molecule, is to show weak fluorescence in an aqueous environment but strong fluorescence in a hydrophobic environment [24]. Thus, it is widely used to detect the disruption of the outer membrane of Gram-negative bacteria [25,26].

**Figure 4:**
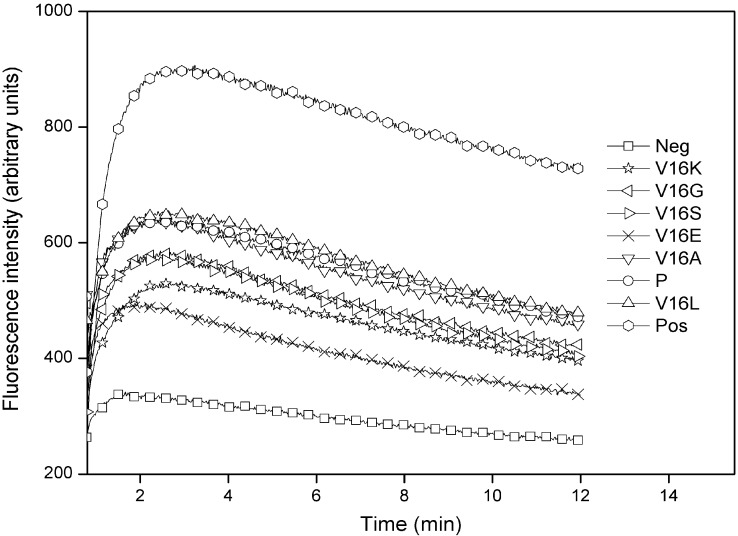
Outer membrane permeabilization induced by peptides as detected by NPN uptake in *E. coli* ATCC 25922. Neg and pos represent the negative control and positive control, respectively.

The outer membrane disturbance was reflected as an NPN fluorescence alteration as shown in Figure 4. It is worth to note that the peptide concentrations used in this assay were lower than the MIC values for most of peptides. The small incremental changes of peptide hydrophobicity caused corresponding florescence changes in the NPN uptake assay, that is, the fluorescence intensity of peptide analogs was correlated with peptide hydrophobicity, which indicated that the outer membrane of Gram-negative bacteria is “sensitive” to the peptide physiochemical parameters. Peptides with higher hydrophobicity exhibited greater outer membrane disturbance on Gram-negative bacteria (Figure 4).

**Figure 5:**
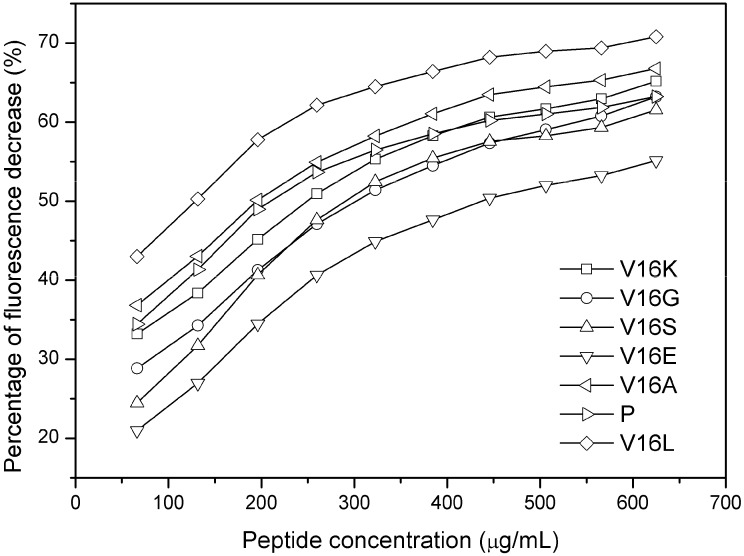
Differential inhibition of dansyl-polymyxin B binding to LPS by peptide analogs.

As most of the outer membrane is LPS, the affinity of peptide analogs to LPS of *E. coli* was further detected by dansyl-polymyxin B displacement assay [27]. As it is the polar face of the molecule that binds LPS, and all the peptides V16A, V16S, V16G and P have the same number of positively charged residues on the polar face, they would be expected to have similar effects on LPS affinity (Figure 5). However, the Lys-substituted peptide exhibited more affinity than the Ser- or Gly-substituted peptides, although V16S and V16G were more hydrophobic than V16K. This result indicates that positive charge plays an important role in the interaction of peptide with LPS.

### 2.4. Membrane Binding

Upon peptide diffusion into the cytoplasmic membrane, evidence of selective interaction between peptides with bacterial *versus* mammalian cytoplasmic membrane was illustrated by the binding events of the peptides to anionic or zwitterionic lipid vesicles. The repositioning of the tryptophan indole group in an environment of reduced polarity upon membrane binding results in a blue shift in the fluorescence excitation maximum coupled with an enhancement of emission intensity. Lipid/peptide (L/P) ratios were maintained high enough at low peptide concentration (2 μM) in order to mimic the initial steps of peptide-membrane binding [28]. The spectra were recorded as shown in Supplementary Figure S1. As shown in Table 4, the Trp emission maxima of the peptide analogs in three environments have been summarized, which were HEPES buffer only, representing an aqueous environment, buffer with PC/PG vesicles mimicking a bacterial cytoplasmic membrane, and buffer with PC/cholesterol vesicles mimicking an eukaryotic cell membrane. The significant blue shifts of Δλ_max_ (17 to 23 nm) for all peptides upon exposure to PC/PG vesicles indicated a membrane partition state of Trp in negatively charged vesicles containing 30% anionic lipids (PG). In contrast, virtually no dramatic shifts in either maximum wavelength or fluorescence intensity were observed for all peptides with PC/cholesterol vesicles. Hence, the peptides in this study showed much stronger specificity against bacteria than eukaryotic cells, which was consistent with the biological activities. 

**Table 4:** Tryptophan fluorescence emission maxima of the peptides in HEPES buffer or in the presence of PC/PG (7:3, w/w) or PC/cholesterol (8:1, w/w) vesicles.

Peptide	HEPES buffer	PC/PG (7:3, w/w) *^a^*	PC/cholesterol (8:1,w/w)
	nm		
V16K	350	329 (21 *^b^*)	349 (1)
V16G	350	329 (21)	349 (1)
V16S	350	328 (22)	349 (1)
V16E	350	333 (17)	349 (1)
V16A	350	328 (22)	349 (1)
P	350	328 (22)	349 (1)
V16L	350	326 (23)	348 (2)

*^a^* Lipid and peptide are in a molar ratio of 50:1. *^b^* Blue shift in emission maximum.

It is noteworthy that the introduction of glutamic acid into the parent peptide (peptide V16E) produced the weakest blue shift (17 nm) upon exposure to anionic lipids, which is strongly consistent with the poorest antibacterial activity of peptide V16E. As aforementioned, the charge interaction of residue E16 with K13 may constrain the flexibility of the peptide molecule by stabilizing the α-helical structure and decrease the net charge of the peptide. Thus, peptides with weaker positive charge and smaller non-polar faces would not insert deeply into biomembranes, as the process depends on the electrostatic interaction and hydrophobic interaction, thus resulting in the loss of antibacterial activity.

In this study, the interactions between peptides and lipids, which were illustrated by NPN assay, dansyl-polymyxin B assay and fluorescence study, are consistent with the previous studies [18,19]. Peptide hydrophobicity and charge play important roles in biological activities. It is also clear that subtle modulation of the sequence of antimicrobial peptides may improve the antimicrobial activity and specificity. The binding results of peptides with the model membranes are consistent with the “membrane discrimination” mechanism [12,13]. 

## 3. Experimental Section

### 3.1. Materials

Rink amide 4-methylbenzhydrylamine resin (0.8 mmol/g) and all *N*-α-Fmoc protected amino acids were purchased from GL Biochem (Shanghai, China). The coupling reagents for peptide synthesis *O*-benzotriazole-1-yl-*N,N,N′,N′*-tetramethyluronium hexafluorophosphate (HBTU), 1-hydroxybenzotriazole (HOBt), *N,N′*-diisopropylethylamine (DIEA), trifluoroacetic acid (TFA), 1-*N*-phenylnaphthylamine (NPN), polymyxin B sulfate, dansyl chloride and lipopolysaccharides (LPS) from *E. coli* 055:B5 were purchased from Sigma (St. Louis, MO, USA). Acetonitrile (HPLC grade) was obtained from Fisher (Hampton, NH, USA). Dichloromethane (DCM), *N,N*-dimethylformamide (DMF), piperidine and 2,2,2-trifluoroethanol (TFE) were analytical grade and purchased Jintai Chemicals, Changchun, China.

### 3.2. Peptide Synthesis and Purification

Peptides were synthesized on solid phase using 9-fluorenyl-methoxycarbonyl (Fmoc) chemistry as described previously [19]. Crude peptides were purified on a Shimadzu LC-6A preparative HPLC system (Shimadzu, Tokyo, Japan) using a Zorbax 300 SB-C_8_ reverse phase column (250 × 9.4 mm inner diameter, 6.5 μm particle size, 300 Å pore size; Agilent Technologies, Santa Clara, CA, USA) with a linear AB gradient at a flow rate of 2 mL/min. Mobile phase A was 0.1% aqueous trifluoroacetic acid (TFA) and B was 0.1% TFA in acetonitrile. The purity of the peptides was verified by analytical RP-HPLC as described below, and the purified peptides were further characterized by mass spectrometry and amino acid analysis.

### 3.3. Analytical RP-HPLC of Peptides

Peptides were analyzed on a Shimadzu LC-20A HPLC using a Zorbax 300 SB-C_8_ column (150 ° 4.6 mm i.d., 5 μm particle size, 300 Å pore size) from Agilent Technologies using a linear AB gradient and a flow rate of 1 mL/min, where solvents A and B were as described above. Absorbance signals of peptides were detected at 210 nm.

### 3.4. CD Spectroscopy

Circular dichroism spectra were measured with a 0.02 cm path length quartz cuvette on a Jasco J-810 spectropolarimeter (Jasco, Tokyo, Japan) at 25 °C. Data were collected from 250 to 190 nm at a sensitivity of 100 millidegrees, response time of 1 s, bandwidth of 1.0 nm and a scan speed of 100 nm/min. Peptides were measured at a concentration of 75 μM in a benign buffer (50 mM KH_2_PO_4_/K_2_HPO_4_, 100 mM KCl, pH 7) or the benign buffer mixed with 50% 2,2,2-trifluoroethanol (TFE). The mean residue molar ellipticities were calculated using the equation [θ] = *θ*/10*lcMn* [29], where *θ* is the ellipticity in millidegrees, *l* is the optical path length of the cuvette in centimeters, *cM* is the peptide concentration in mole/liter and *n* is the number of residues in the peptide.

### 3.5. Measurement of Antibacterial Activity

Minimal inhibitory concentrations (MIC) were determined using a broth dilution method [30]. Briefly, bacteria were grown overnight at 37 °C in Mueller–Hinton (MH) Broth, diluted in the same medium and transferred into 96-well microtiter plates (90 μL/well). Peptides were serially diluted using 0.2% bovine serum albumin containing 0.01% acetic acid and added to the microtiter plates in a volume of 10 μL per well to give a final concentration of 5 × 10^5^ CFU/mL. MICs were defined as the lowest peptide concentration that inhibited bacterial growth after incubation for 24 h at 37 °C.

### 3.6. Measurement of Hemolytic Activity

Peptide samples were serially diluted using phosphate-buffered saline (PBS) in 96-well plates (round-bottom) to give a volume of 60 μL of the sample solution in each well. Human erythrocytes that had been anticoagulated using EDTAK_2_ were collected by centrifugation (1,000 *×g*) for 5 min, washed twice with PBS, then diluted to a concentration of 2% in PBS. The erythrocytes (60 μL of 2% solution) were added to each well to achieve a final concentration of 1% human erythrocytes per well, and the reactions were incubated at 37 °C for 4 h. The plates were then centrifuged for 10 min at 3000 rpm and the supernatant (80 μL) was transferred to a 96-well plate (flat-bottom). The release of hemoglobin was determined by measuring the absorbance of the supernatant at 540 nm. Peptide samples were diluted in a 2-fold series to determine the minimum concentration at which hemolysis occurred. Erythrocytes in PBS and distilled water were used as controls of 0% and 100% hemolysis, respectively. Hemolysis percentage was calculated by the absorbance of peptide at the concentration of 1,000 μg/mL and compared with the 100% hemolysis.

### 3.7. Permeabilization of Bacterial Outer Membranes

The NPN uptake assay was used to detect disturbances in the outer membrane of Gram-negative bacteria by the peptides [31]. An overnight culture of *E. coli* (1 mL, ATCC 25922) was transferred into 50 mL of MH broth. After incubation for 2–3 h, middle midlog phase cells (OD600 = 0.4–0.6) were washed once and resuspended to an OD600 of 0.5 (5 × 108 CFU/mL) with 5 mM HEPES buffer containing 5 mM NaN_3_ (pH 7.4). Fluorescence measurements were run on a Shimadzu RF-5301 spectrofluorometer using a 3 mL quartz cuvette with an excitation wavelength of 350 nm and an emission wavelength of 420 nm at 25 °C. The cell suspension (2 mL) in the cuvette was first measured for 0.5 min, then 20 μL of NPN solution in acetone (0.5 mM) was added and the results were recorded for about 15 s. Immediately following this step, 20 μL of peptide solution in water was added to the cuvette and the reaction cuvette was monitored for 10 min. Assays with no peptide added or with polymyxin B sulfate (final concentration 0.64 μg/mL) were used as the negative and positive controls, respectively.

### 3.8. Dansyl-Polymyxin B Displacement Assay

Dansyl-polymyxin B was synthesized, purified and quantified as reported [32]. It was then dissolved in HEPES buffer (5 mM, pH 7.2) at the concentration of 100 μM and the final concentration of LPS is 3 μg/mL in the same buffer. Fluorescence measurements were run on a Shimadzu RF-5301 spectrofluorometer using a 3 mL quartz cuvette with an excitation wavelength of 350 nm and an emission wavelength of 420 nm at 25 °C. For displacement assay, 15 μL of peptide solution (10 mg/mL) was titrated into the cuvette containing 2 mL LPS and 0.25 mL dansyl-polymyxin B, and the decrease in the observed fluorescence (percent inhibition) was recorded. 

### 3.9. Preparation of Lipid Vesicles

Unilamellar vesicles were prepared by a standard procedure with either PC/PG (7:3, w/w) or PC/cholesterol (8:1, w/w) as described previously [33]. Briefly, the desired mixtures of lipids were dissolved in a chloroform/methanol mixture (2:1, v/v), dried under nitrogen and then lyophilized for 24 h to remove traces of organic solvent. Dry lipid films were suspended in 10 mM HEPES buffer containing 150 mM NaCl (pH 7.4) by vortex mixing. After five freeze-thawing cycles, the suspension was extruded 15 times through double polycarbonate membranes with 0.1 μm diameter pores on an Avanti mini-extruder apparatus. The lipid concentration was determined by phosphorus analysis as previously reported [34].

### 3.10. Fluorescence Spectroscopy

Fluorescence emission spectra of peptide Trp residues were monitored by a Shimadzu RF-5301 spectrofluorometer. The excitation of wavelength was set as 280 nm and the emission spectra of each peptide, with or without lipid vesicles, were recorded between 300 and 450 nm. Thus three curves were recorded for each peptide, in HEPES buffer or with either PC/PG vesicles or PC/cholesterol vesicles. The blue shift of Trp residues in vesicles were referred to in buffer condition. The final concentrations of peptide and lipid were 2 μM and 100 μM (1/50), respectively.

## 4. Conclusions

In this study, the activity of antimicrobial peptides with different hydrophobicity were investigated against Gram-negative bacteria. In general, peptide antimicrobial activity and hemolytic activity correlated with peptide hydrophobicity. Peptide membrane permeabilization was also correlated with peptide hydrophobicity. Positive charges may enhance the interactions of peptides with the bacterial outer membrane through LPS binding. The peptides in this study showed great antimicrobial activity against Gram-negative bacteria and weak hemolytic activity against human red blood cells. A single amino acid substitution approach may be valuable for the *de novo* design of antimicrobial peptides with enhanced activity.

## Supplementary Materials

Supplementary materials can be accessed at: http://www.mdpi.com/1420-3049/19/8/10803/s1.

## Supplementary Files

Supplementary File 1
