# Supplementary material for: Effects of Single Amino Acid Substitution on the Biophysical Properties and Biological Activities of an Amphipathic α-Helical Antibacterial Peptide Against Gram-Negative Bacteria
Source: Molecules. 2014 Jul 24;19(8):10803–17. doi: 10.3390/molecules190810803 (PMC6271477; doi:10.3390/molecules190810803)

Supplementary File

Figure S1. Trp fluorescence emission spectrum of 2 μM peptide analogs upon interacting without (triangles) or with vesicles composed of PC:PG (7:3, w/w) (squares) or PC/cholesterol (8:1, w/w) (circles).

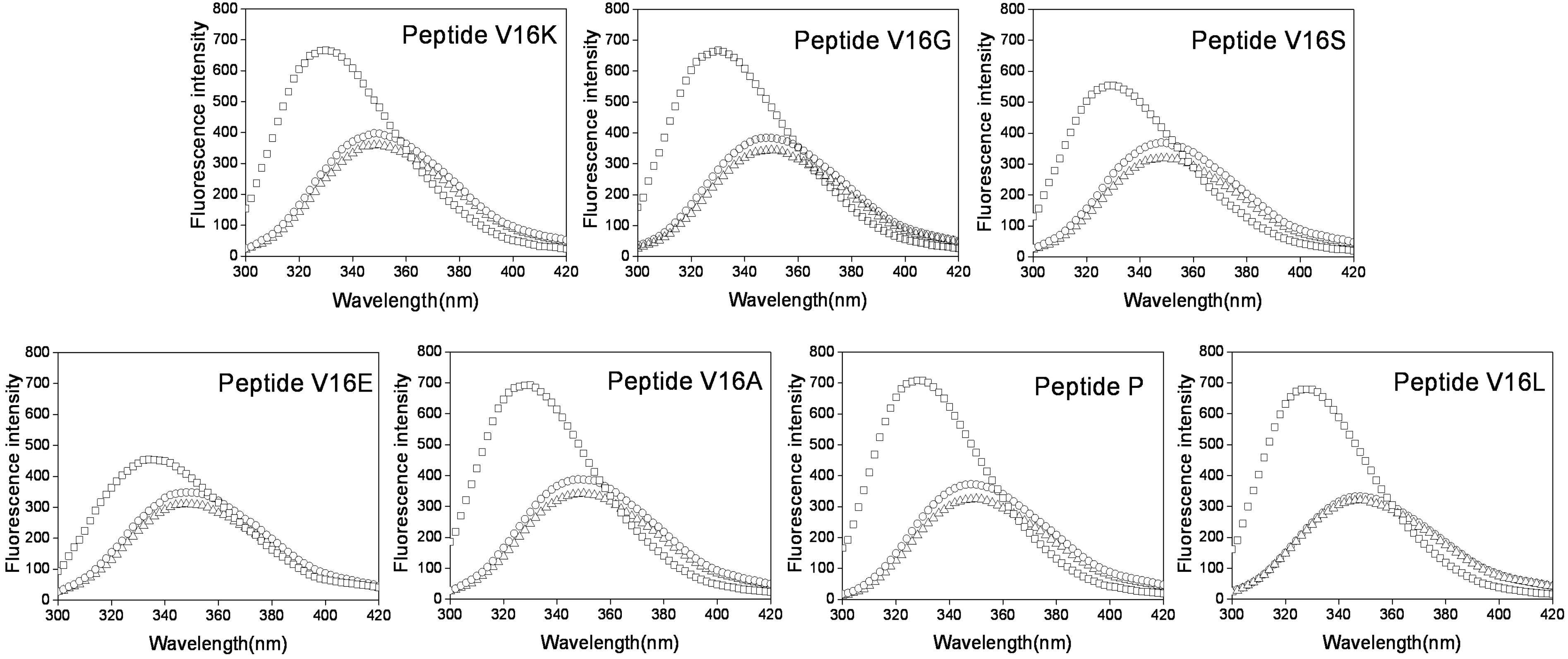

Supplement: Supplementary File 1 [file molecules-19-10803-s001.pdf]
